# Platelet count as a biomarker for monitoring treatment response and disease recurrence in recurrent epithelial ovarian cancer

**DOI:** 10.1186/s13048-020-00682-z

**Published:** 2020-07-18

**Authors:** Qinghong Hu, Abha Hada, Liping Han

**Affiliations:** 1 Department of Radiation Oncology, The First Affiliated Hospital of Zhengzhou University, Zhengzhou, Henan 450002 People’s Republic of China; 2 B.P. Koirala Institute of Health Sciences, Sunsari, Dharan, Nepal; 3 Department of Obstetrics and Gynecology, The First Affiliated Hospital of Zhengzhou University, No.1, Jianshe East Road, Zhengzhou, Henan 450002 P.R. China

**Keywords:** Platelet count, D-dimer, Fibrinogen, Recurrent EOC, Prognosis, Apoptosis

## Abstract

**Objectives:**

We sought to determine the impact of pretreatment plasma platelet levels, dimerized plasmin fragment (D-dimer) and fibrinogen in recurrent epithelial ovarian cancer (EOC) and the impact of platelet levels on SKOV3 cell lines growth and responsiveness to chemotherapy.

**Methods:**

Under approval of ethical committee, we identified 104 women with recurrent EOC who underwent treatment between January 2010 and February 2015. Reviewing clinical, laboratory, and pathologic records from this retrospective cohort, we analyzed the correlation between pretreatment plasma D-dimer, fibrinogen, platelet levels and clinicopathological parameters, progression free survival (PFS) and overall survival (OS). Inco-culture experiments human ovarian cancer SKOV3 cell lines were used to test the effect of platelet levels on tumor growth and responsiveness to docetaxel.

**Results:**

Of the 104 recurrent EOC, thrombocytosis at diagnosis and the decrease of platelet count by less than 25% after primary therapy were associated with worse median progression free survival (*P* = 0.003;*P* = 0.021) and median overall survival (*P* = 0.009;*P* = 0.009). Mean platelet levels declined at the end of primary therapy(*P* < 0.001) and rose at recurrence(*P* = 0.007). In multivariate analysis, elevated platelet levels at primary therapy and the decrease of platelet count less than 25% after primary therapy were unfavorable prognostic factor for PFS(*P* = 0.022; *P* = 0.015) and OS(*P* = 0.013;*P* = 0.007) in recurrent EOC, but elevated plasma D-dimer and fibrinogen were not. In SKOV-3 ovarian cancer cell lines, suitable concentration platelet co-culture protected against apoptosis (*P* < 0.05).

**Conclusions:**

Platelet count during treatment could be used as a biomarker used for monitoring the disease recurrence and predicting treatment response. And platelet with suitable concentration co-culture protected against apoptosis in SKOV3 cell line, which may explain clinical observations.

## Introduction

Malignant tumors are frequently accompanied by increased risk of hematological abnormalities. Elevated plasma D-dimer, fibrinogen, and platelet levels have been found in various malignancies [1] and are correlated with poor clinical outcome [2, 3], while some do not find any correlation [4, 5]. Platelets are associated with metastasis, angiogenesis, epithelial-to-mesenchymal transitio and tumor cell proliferation [6]. Pretreatment thrombocytosis in EOC patients is closely associated with more malignant disease phenotype and poorer prognosis [7]. Thrombocytos is may independently predict the diseases pecific survival for EOC patients. Thrombocytosis, accompanied by increasing of platelet aggregation rates, is associated with more aggressive tumor biology in EOC [8]. Some evidence indicates that tumor growth is incubated by a paraneoplastic thrombocytosis in a paracrine circuit by thrombopoeitic cytokines in ovarian cancer culture models [9]. Ovarian cancer is ranked fifth of ten leading cancer types based on the estimated new cancer cases and deaths in woman in the latest statistics [10]. Although there is a high rate of remission for advanced disease after the main treatment with surgery and chemotherapy, recurrence is common in patients with over 60% of advanced stage [11]. Despite the available literature regarding elevated plasma D-dimer, fibrinogen, and platelet levels at the time of primary treatment, it is unclear if this three at the time of recurrence have clinical significance. We considered whether plasma D-dimer, fibrinogen, and platelet levels could serve as biomarkers, like CA125, to monitor treatment response and to follow up during the surveillance for recurrence of the EOC. In this study, we evaluate the association between pretreatment plasma D-dimer, fibrinogen, platelet levels, survival, and other clinic-pathologic factors in women with recurrent EOC and the impact of platelet levels on SKOV3 cell lines growth and responsiveness to chemotherapy.

## Methods

### Patients

Under an ethical committee-approved protocol of the First Affiliated Hospital of Zhengzhou University, we identified 104 women with recurrent EOC who underwent treatment between January 2006 and February2015 in our hospital. The inclusion criteria for this study were as follows: Patients with (1) pathological diagnosis of EOC, (2) treatment with surgical cytoreduction performed by a gynecologic oncologist in addition to taxane and/or platinum-based chemotherapy between January 2010 and February 2015 in our hospital, and (3) adequate clinical information in the medical record. Patients were excluded from the study for the following criteria: (1) did not receive primary therapy or follow-up at the institution of record; (2) no recurrence of disease;(3) history of other malignancies, myeloproliferative disease, inflammatory disease, splenectomy, or other confounding causes of thrombocytosis, and (4) known congenital thrombophilia, ongoing anticoagulant treatment, pregnancy, or stroke or neurosurgery within 6 months.

### Measurements of plasma D-dimer, fibrinogen, platelet levels and CA125

Clinical data collected included patient demographics, tumor characteristics, details of treatment, and outcome data. Pre-treatment plasma D-dimer, fibrinogen, platelet levels and CA125 measurements were recorded. Then platelet levels and CA125 measurements were recorded after the completion of surgery, 6 cycles of cytotoxic chemotherapy, after treatment completion and at the time of diagnosis of recurrence. In this cohort of patients, thrombocytosis was considered to be a platelet count ≥300,000 cells/μl, hyperfibrinogenemia was defined as plasma fibrinogen level above 4 g/l, high plasma D-dimer level was considered based on the standard cutoff level of 0.3 mg/l, consistent with published criteria [3, 10]. Postoperative gross residual disease of < 1 cm was classified as microscopic. Conversely, residual disease > 1 cm gross was defined as macroscopic. The percent of change in platelet counts after treatment compared to the counts pretherapy were calculated. To reduce the effect of treatment-induced myelosuppression, the platelet count was determined 14 days after the end of the chemotherapy and the ratio of platelet count before and after treatment was used for further analysis.

### Pre-clinical analysis

#### Cell lines and culture condition

The derivation of the human ovarian cancer cell lines SKOV3 was purchased from American type culture collection (ATCC) cell library. The cell lines were maintained in RPMI-1640 with 10% fetal bovine serum. Cell lines were routinely genotyped to confirm identity and tested to confirm absence of Mycoplasma. Cells were maintained at 37 °C in a humidified incubator infused with 20% O2 and 5% CO2.

#### Docetaxel

Docetaxel (Aventis Pharma S.A.) was obtained from surplus clinical samples from the clinical pharmacy associated with Intravenous Dispensing Center of the first affiliated hospital of Zhengzhou University.

#### Platelet isolation for in assays

Platelets were prepared for in vitro assays in a manner that would remove plasma contents and nucleated cells. Whole blood was drawn from clinical volunteers into a syringe pre-loaded with 1:9 v/v 3.8% sodium citrate and mixed 1:1 v/v with tyrodes buffer lacking Mg^2+^ and Ca^2+.^ Blood was centrifuged at1000rpm for 10 min, at 22 °C. The platelet-rich plasma fraction was passed through a filtration column of Sepharose 2B beads (Sigma Aldrich, St Louis,MO) loaded into a 10 ml disposable plastic syringe with a 40 μm nylon net filter at the bottom and sepharose 2B beads previously washed in acetone 1:1 v/v, followed by 0.9% NaCl 1:1 v/v, and Buffer 1:1 v/v. Platelets were counted with a hemocytometer by phase-contrast microscopy at 400× magnification and concentration was adjusted to 1 × 10^9^/ ml by platelet buffer (Glucose 5.5 mmol/L, Tris, 15 mmol/L, NaCl 0.14 mmol/L, pH 7.4).

#### Potential effects of platelets on apoptosis and response to chemotherapy in assays

To examine potential effects of platelets on apoptosis and response to chemotherapy, we incubated cancer cells with platelets using a tissue co-culture system and observed consistent protection against apoptosis. To assess the effect of platelets on apoptosis, cells were plated in 6-well plates at 50,000 cells per plate. At 50% confluence, media was changed to serum-free for 24-h prior to starting treatment. After serumstarvation, platelets were isolated and added to achieve a final dose of 1 × 10^8^ platelets/mL. Docetaxel was dosed at 5 nM based on previously published IC50 levels. Controls utilized an equivalent volume of the appropriate buffer. All treatments were performed in triplicate. After 72 h of platelet and docetaxel exposure, cell viability was assessed using Annexin V-FITC and PropidiumIodide (PI) staining (BD Pharmingen TM) by flow cytometry. In vitro experiments were performed as described. Internal controls (*n* = 3) were performed for each experiment given the variability in baseline apoptosis rates seen between experiments in order to avoid batch error.

#### Platelet density on apoptosis and response to chemotherapy

To test the effect of platelet density on apoptosis and response to chemotherapy, cells were plated in 6-well plates at 50,000 cells per plate. At 50% confluence, media was changed to serum-free for 24-h prior to starting treatment. After serumstarvation, platelets were isolated and added to achieve a final dose of 0.5 × 10^8^ platelets/mL,1 × 10^8^ platelets/mL,2 × 10^8^ platelets/mL,4 × 10^8^ platelets/mL. Docetaxel was dosed at 5 nM based on previously published IC50 levels. Controls utilized an equivalent volume of the appropriate buffer. All treatments were performed in triplicate. After 72 h of platelet and docetaxel exposure or not, cell viability was assessed using Annexin V-FITC and Propidium Iodide (PI) staining (BD PharmingenTM) by flow cytometry. In vitro experiments were performed as described. Internal controls (*n* = 3) were performed for each experiment given the variability in baseline apoptosis rates seen between experiments in order to avoid batch error.

### Statistical analyses

All the categorical variables were analyzed by chi-square test and ANOVA test for continuous variables. Kaplan-Meier curves were used to estimate the distribution of PFS and OS, and log-rank test was performed to compare the difference between the survival curves. Variables, which were identified as statistically significant in univariate analysis, were included in the multivariate survival analysis using the Cox proportional hazard model. Progression-free survival (PFS), interval to progression, was defined as the time from the conclusion of six cycles of primary therapy to the time when the clinical diagnosis of recurrence by physical exam, laboratory evaluation, and/or imaging was reported, whichever occurred first. Overall survival (OS) was defined as the time from the conclusion of six cycles of primary chemotherapy to the time of last follow-up or death. Survival times of patients who were known to be alive and who were dead as a result of other causes were censored with the last follow-up date. All statistical analyses were performed using SPSS 17.0 statistical software. *P* values < 0.05 were considered of statistical significance.

## Results

### Patient clinical characteristics

Of the 104 women with recurrent EOC, 77.9% had advanced stage (III or IV), 76.9% had high-grade disease, 50.0% had middle or grade serous subtype and 57.7% had thrombocytosis at the time of diagnosis. The median age was 53 years (range 37–81 years) and the mean platelet level was 299.8000 /μl (range 108,000–545,000 cells/μl) at diagnosis.

### Relationship between clinicopathological parameters and pretreatment plasma D-dimer/fibrinogen/platelet levels and serum CA125

Plasma D-dimer and fibrinogen levels pretreatment were above the normal value in 29(27.9%) and 90(86.5%) patients, respectively. The incidence of thrombocytosis was 57.7% (60 /104). The incidence of elevated serum CA125 levels in 104 patients with recurrent ovarian cancer was 97.1% (101 /104). Here we found that elevated platelet levels were significantly associated with FIGO stage (*P* = 0.021) and histological type was associated with elevated plasma D-dimer levels (*P* = 0.016), but not with other clinicopathological parameters. (Table 1).

**Table 1:** Relationship between clinicopathological parameters and different levels of plasma D-dimer, fibrinogen and serum CA125 in 104 patients with recurrent EOC

		Platelet count 1000cells/μl			Plasma Fibrinogen g/l			Plasma D-dimer mg/l			serum CA125 U/ml		
Variable	*n*	≤300	> 300	*P*	2–4	> 4	*P*	≤0.3	> 0.3	*P*	≤35	> 35	*P*
Age, y				0.340			0.386			0.566			0.685
≤ 55	39	28	21		27	12		5	34		1	38	
> 55	65	26	39		48	17		9	56		2	63	
Stage				0.335			0.473			0.373			0.531
I or and II	23	11	12		16	7		4	19		1	22	
III or and IV	81	33	48		59	22		10	71		2	79	
Histological type				0.432			0.016			0.402			0.324
Serous	64	28	36		41	23		6	58		1	63	
Non serous	40	16	24		34	6		8	32		2	38	
Grade				0.021			0.273			0.410			0.549
Low	24	15	9		19	5		4	20		1	23	
High	80	29	51		56	24		10	70		2	78	
Postoperative residual disease				0.069			0.514			0.402			0.301
Microscopic	66	32	34		48	18		8	58		1	65	
Macroscopic	38	12	26		27	11		6	32		2	36	

*CI* confidence interval, *HR* hazard ratio

### Factors affecting progression free survival and overall survival: by univariate and multivariate analyses

**Fig. 1:**
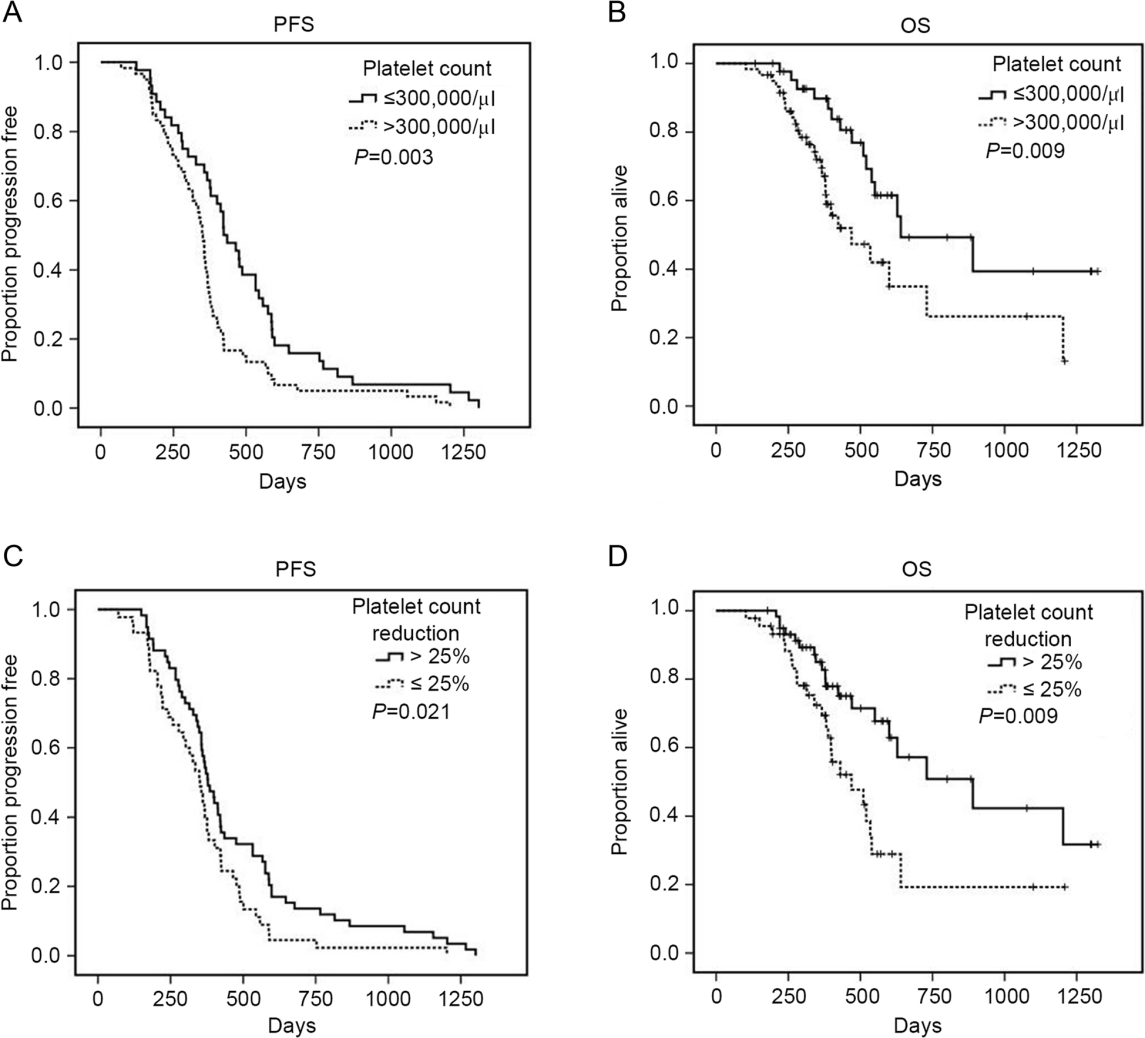
Cumulative survival curves for PFS and OS according to pretreatment platelet count and the reduction of platelet count by Kaplan-Meier method in the 104 patients with recurrent EOC. **a** Progression free survival for pretreatment platelet count (*P* = 0.003); **b** Overall survival for pretreatment platelet count (*P* = 0.009); **c** Progression free survival for the reduction of platelet count (*P* = 0.021); **d** Overall survival for the reduction of platelet count (*P* = 0.009)

Of the 104 women with recurrent EOC, pretreatment thrombocytosis was associated with worse median progression free survival (11.6 vs.14.1 months, *P* = 0.003, Fig. 1a) and median overall survival (15.6vs. 21.3 months, *P* = 0.009, Fig. 1b). However, elevated plasma D-dimer and fibrinogen were not confirmed in univariate analysis and multivariate analysis (Tables 2 and 3). Next, we investigated the association between the percentage of reduction of platelet count relative to the pretreatment level and patient survival. Intriguingly, we found that a reduction less than 25% in platelet count post-treatment was associated with poor PFS and OS (*P* = 0.021, *P* = 0.009, Fig. 1c and d). After adjustment for tumor stage, histological grading, and residual disease after cytoreductive surgery, multivariate analysis rendered decrease of platelets to a cut-off value of less than25% reduction after treatment as an independent, unfavorable prognostic factor for PFS. (Tables 2 and 3).

**Table 2:** Univariate and multivariate analysis of prognostic factors in progression free survival

	Univariate analysis				Multivariate analysis			
Variable	HR	95.0% CI		*P* value	HR	95.0% CI		*P* value
Age (y) ≤55 vs. > 55	1.351	0.903	−2.020	0.143	–	–	–	–
Postoperative residual disease Microscopic vs. Macroscopic	3.637	2.376	−5.566	*P <* 0.001	2.761	1.752	−4.351	*P <* 0.001
Grade Low vs. High	2.499	1.535	−4.067	*P <* 0.001	1.912	1.141	−3.202	0.014
Stage I or II vs. III or IV	2.344	1.360	−4.039	0.002	1.592	0.883	−2.872	0.122
Platelet count (1000/μl) ≤300 vs. > 300	1.820	1.214	−2.728	0.004	1.626	1.073	−2.466	0.022
Platelet count reduction > 25% vs. ≤25%	1.594	1.068	−2.378	0.023	1.651	1.100	−2.478	0.015
Plasma fibrinogen(g/l) 2–4 vs. > 4	0.914	0.592	− 1.411	0.685	–	–		–
Plasma D-dimer (mg/l) ≤0.3 vs. > 0.3	0.894	0.507	−1.574	0.697	–	–	–	–

*CI* confidence interval, *HR* hazard ratio

**Table 3:** Univariate and multivariate analysis of prognostic factors in overall survival

	Univariate analysis				Multivariate analysis			
Variable	HR	95.0% CI		*P* value	HR	95.0% CI		*P* value
Age (y) ≤55 vs. > 55	1.606	0.844	−3.058	0.149	–	–	–	–
Postoperativeresidual disease Microscopic vs. Macroscopic	5.811	3.019	−11.182	*P <* 0.001	4.300	2.124	−8.703	*P <* 0.001
Grade Low vs. High	2.695	1.225	−5.928	0.014	1.714	0.733	−4.004	0.214
Stage I or II vs. III or IV	2.972	1.195	−7.393	0.019	1.799	0.656	−4.930	0.254
Plateletcount (1000/μl) ≤300 vs. > 300	2.298	1.208	−4.373	0.011	2.363	1.202	−4.645	0.013
Platelet count reduction > 25% vs. ≤25%	2.222	1.200	−4.113	0.011	2.363	1.262	−4.423	0.007
Plasmafibrinogen(g/l) 2–4 vs. > 4	1.092	0.573	−2.083	0.789	–	–	–	–
Plasma D-dimer (mg/l) ≤0.3 vs. > 0.3	0.712	0.315	−1.608	0.413	–	–	–	–

*CI* confidence interval, *HR* hazard ratio

### Platelet count and CA125 trends: during treatment and until the clinical diagnosis of recurrence

**Fig. 2:**
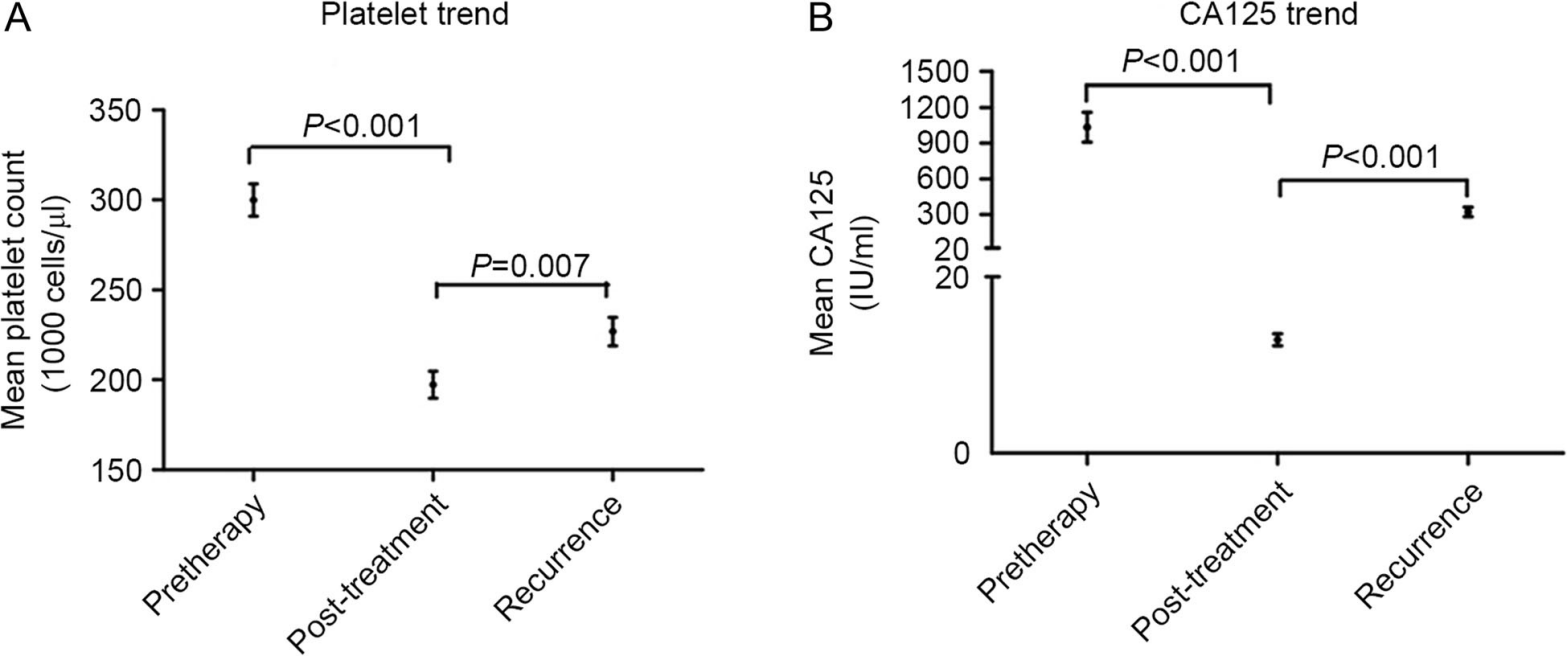
**a** Platelet counts trend during the course of therapy and at the time of disease recurrence in 104 patients of recurrent EOC; **b** CA125 levels trend during the course of therapy and at the time of disease recurrence in 104 patients of recurrent EOC; The platelet counts and CA125 levels are given in median with SD

To consider platelet and CA125 trends, data of 104 patients was recorded through primary diagnosis, primary treatment and until the clinical diagnosis of recurrence. Of the patients with recurrent EOC (*n* = 104),86.5%patients had a normal platelet count (mean 197,000 cells/μl) after primary therapy,and mean platelet counts at the diagnosis of recurrence were found to be increased to 227,000 cells/μl compared to that after primary therapy (*P* = 0.007, Fig. 2a).CA125 is a standard tumor marker followed in ovarian cancer to track the efficacy of primary therapy and in surveillance for recurrence. The mean CA125 level at diagnosis was 1034.8 IU/mL (normal < 35 IU/mL). In contrast, 81.4%had a normal CA125 level at the conclusion of primary therapy with a mean 75.1 IU/mL, and the mean post-treatment CA125 level was 12.8 IU/mL. At the clinical diagnosis of disease recurrence, CA125 was elevated in 80.8% of patients, with a median 323.1 IU/mL compared to that after treatment (*P* < 0.001, Fig. 2b). Among patients with a CA125 < 35 IU/mL at the time of recurrence, mean platelet levels at the diagnosis of recurrence were increased to 249.700 cells//μl compared to that (197.100 cells/μl) at the conclusion of primary therapy (*P* = 0.046).

### Normalization of platelet counts post-treatment is associated with disease response to therapy

In the patients with recurrent EOC who experienced a compete response to therapy > 6 months, 56.0% (51/91) had thrombocytosis at diagnosis, and 92.3% (84/91) of these patients had normalized platelet levels by the end of primary therapy. In the treatment resistant cohort (*n* = 13) who experienced a complete response to primary therapy that was durable for < 6 months, 76.9% (10/13) patients had thrombocytosis at beginning of primary therapy. At the end of primary therapy, only 46.2% (6/13) had normalized platelet counts. These data indicate a correlation between the normalization of platelet counts at the end of primary therapy and disease response to therapy (*P* < 0.001)*.*

### Platelets resistance against chemotherapy-induced apoptosis

**Fig. 3:**
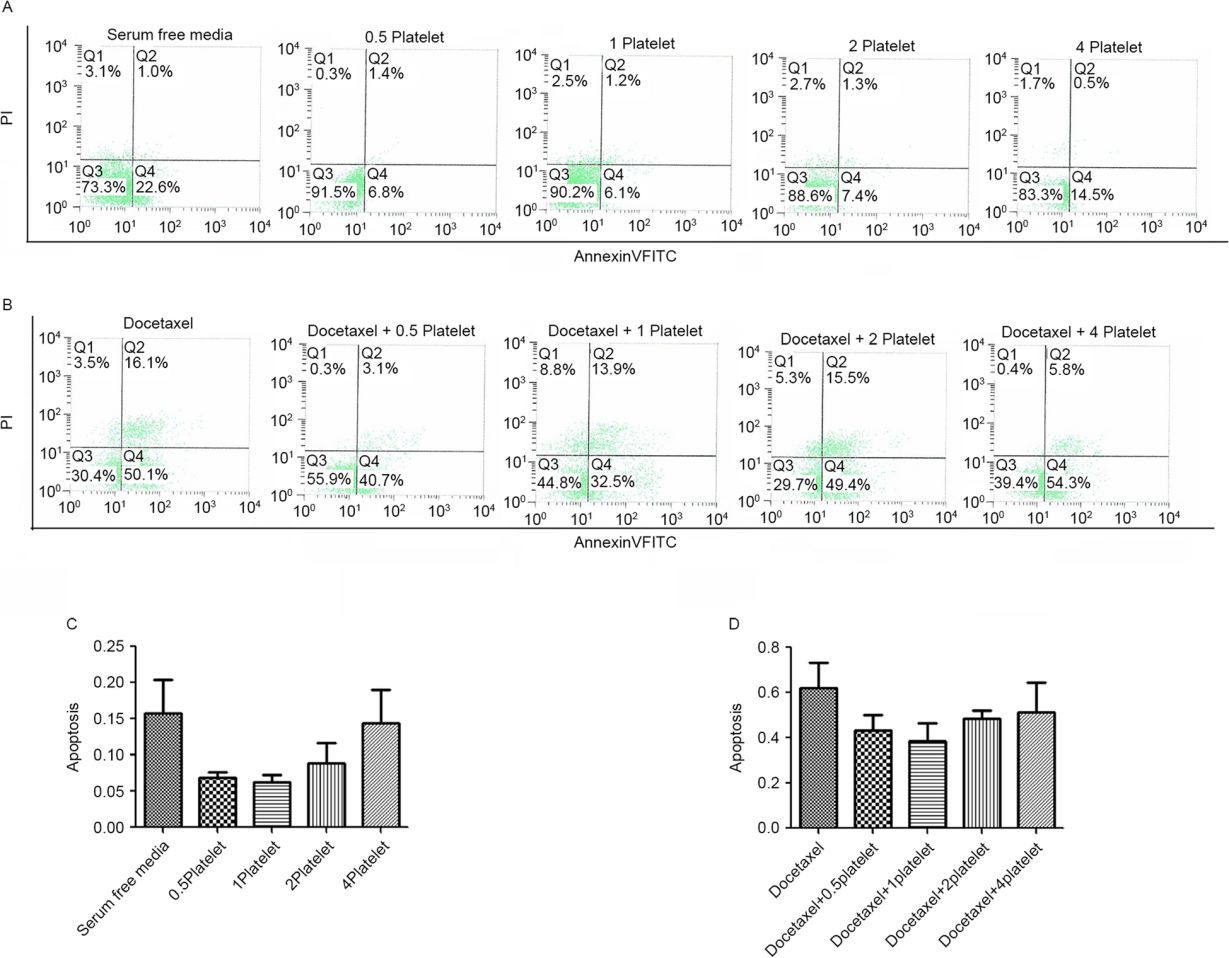
**a** Apoptosis rates of SKOV-3 co-cultured with platelets 0.5 × 10^8^/mL,1 × 10^8/mL,2 × 10^8^/mL,4 × 10^8^/mL compared to serum free media; **b** With the addition of 5 nM docetaxel, apoptosis rates of SKOV-3 co-cultured with platelets 0.5 × 10^8^/mL,1 × 10^8/mL,2 × 10^8^/mL,4 × 10^8^/mL compared to only 5 nM docetaxel; **c** SKOV-3 co-cultured with platelets 0.5 × 10^8^/mL,1 × 10^8^/mL,2 × 10^8^/mL,4 × 10^8^/mL, platelets decreased apoptosis from 15.7 to 7.0%(*p* = 0.01),6.3%(*p* = 0.008),8.8%(*p* = 0.044),14.4%(*p* = 0.706) compared to serum free media respectively; **d** With the addition of 5 nM docetaxel, platelets 0.5 × 10^8^/mL,1 × 10^8^/mL,2 × 10^8^/mL,4 × 10^8^/mL decreased apoptosis from 61.9 to 43.2%(*p* = 0.03),38.6% (*p* = 0.015),48.6%(*p* = 0.098),51.1% (*p* = 0.26) compared to only 5 nM docetaxel respectively

Tissue co-culture with platelets demonstrated consistent protection against apoptosis with and without exposure to docetaxel. Platelet activation was evident by the aggregation of platelets within the initial hours of 37°Cincubation. Incubation of SKOV3 cells with platelets in serum-free conditions reduced apoptosis 59.9% (*p* = 0.008). After incorporating docetaxel, incubation of the SKOV-3 cell line with platelets reduced apoptosis by 37.6% (*p* = 0.0015). (Fig. 3b and d).

We next compared the apoptotic rates of different platelet concentrations and tumor cells. To observe these changes in apoptotic rates, ovarian cancer cells were incubated with different concentrations of platelets for 72 h in a serum free environment with and without docetaxel 5 nM. Incubation of SKOV3 cells with platelets of the dose 0.5 × 10^8^platelets/mL,1 × 10^8^platelets/mL,2 × 10^8^ platelets/mL,4 × 10^8^platelets/mL in serum-free conditions reduced apoptosis by 55.4%(*p* = 0.01),59.9%(*p* = 0.008),43.9%(*p* = 0.044),8.3%(*p* = 0.706), respectively (Fig. 3a and c). After incorporating docetaxel, incubation of theSKOV3cellwith platelets of the dose 0.5 × 10^8^platelets/mL,1 × 10^8^ platelets/mL,2 × 10^8^platelets/mL,4 × 10^8^platelets/mL reduced apoptosis by 30.2%(*p* = 0.03),37.6% (*p* = 0.015),21.5%(*p* = 0.098),17.4% (*p* = 0.26), respectively (Fig. 3b and d). These data suggest that platelets have an anti-apoptotic effect on SKOV3 cell, and they suggest that the anti-apoptotic performance of platelets in vitro was comparable at the different seeding density. Since the culture of the SKOV3 cell with platelets incubated with 4 × 10^8^platelets/mL did not reduce a significantly greater number of apoptosis rates than that with 1 × 10^8^platelets/mL, the lower seeding density was preferable.

## Discussion

Much of the research in recent years has examined the prognostic value of pretreatment platelet count in ovarian cancer [12, 13]. Notably, most of the studies have investigated the impact of the platelet count at the time of diagnosis. To our knowledge, this is the first and the largest study to evaluate the significance of plasma D-dimer, fibrinogen and platelet levels on prognosis of recurrent epithelial ovarian cancer during the course of therapy and at the time of recurrence. Also this is the first study to evaluate predictive significance of the percent of reduction of platelet level at the end of primary therapy on interval to progression and overall survival of recurrent epithelial ovarian cancer. And in this study, the result of SKOV-3 ovarian cancer cell lines co-culturing with suitable concentration platelets provides further evidence supporting a plausible role for platelet in aggressive ovarian cancer biology.

It is increasingly recognized that multiple biological components participate in a cooperative relationship between the platelets and malignancies. Cross-talk between platelets and tumor cells has been shown to participate in tumor hemorrhage [14], the epithelial-to-mesenchymal transition which promotes invasive behavior and extravasation of tumor cells, metastasis by shielding the tumor cells against NK cell mediated tumor lysis, as well as arrest of tumor cells for metastasis in target tissues [15–17]. Platelets have been shown to sequester angiogenesis regulators in addition to other mitogens and release these compounds from alpha-granules in a manner that modulates angiogenesis [18].

Previous studies from different patient cohorts have showed that elevated platelet is a poor prognostic factor [9, 19]. Here we have demonstrated through a cohort of patients with disease recurrence that elevated platelet count before treatment is associated with shortened interval to progression and decreased overall survival, but elevated plasma D-dimer and fibrinogen level were not found to be significant, independent prognostic marker for recurrentepithelial ovarian cancer by univariate and multivariate analyses. Interestingly, here we found with strong evidence that the percent of reduction of platelet level at the end of primary treatment is an independent predictive factor of treatment response and the inadequate reduction of the platelet count is associated with shortened interval to progression and decreased overall survival of the recurrentepithelial ovarian cancer. The majority of patients with advanced stage ovarian cancer will develop recurrence. Consideration for the reduction of platelet level of the recurrentepithelial ovarian cancer patients may also help identify those who will benefit from targeted treatment. Targeting platelet counts variation tendency in ovarian cancer patients may represent a viable therapeutic target for the recurrentepithelial ovarian cancer.

The fact that there is fluctuation of platelet count during the course of primary treatment suggests its role in prediction of tumor recurrence and dependence of tumor progression. Moreover, it have been found that at the time of disease recurrence, an increased platelet count is present and was confirmed by our observations [9, 19, 20]. Platelet count is probably directly dependent on the amount of tumor cells [9] and bone marrow may be adversely affected by adjuvant chemotherapy as a result of which patients may be less likely to genrate thrombocytosis in the setting of post-treatment. Disease recurrence may represent chemoresistance or altered cancer cells that have become more aggressive compared to the primary tumor. This may explain why platelet would increase at recurrence [21].

Chemoresistance is a common occurrence in recurrent EOC and platelets may contribute to this. Here we observe that elevated platelet counts at the end of primary therapy are associated with higher rates of relapse and lower rates of response to chemotherapy and the normalization of platelet counts is associated with the disease response to the primary therapy. Chemotherapy was found to be more effective in the context of thrombocytopenia in breast cancer model [22, 23] and platelets seemed to confer resistance to apoptosis induced by taxane chemotherapy [24, 25]. Pretreatment thrombocytosis and reduction of platelet level at the end of primary treatment correlate with worsened overall survival, suggesting that variation trend of platelet may be specific to the types of therapy used. CA125 is a standard tumor marker followed in ovarian cancer to track the efficacy of primary therapy and in surveillance for recurrence [26]. We demonstrated that pre-therapy platelet counts and reduction of platelet level at the end of primary treatment might be useful as a tumor marker, as CA125 levels, to monitor treatment response and during surveillance for recurrence.

The relationship between elevated platelets and malignancy signifies the potential role of antiplatelet drugs in the treatment of patients of EOC with thrombocytosis. There is evidence suggesting that anticoagulants may alter the potential of platelets to facilitate angiogenesis by decreasing the release of vascular endothelial growth factors [27]. A series of studies have indicated a visibly reduced malignancy risk in individuals treated with low-dose aspirin [24, 28–32]. Nevertheless, these data need confirmation through further investigation. Based on our observation in patients with recurrent epithelial ovarian cancer,we hypothesized and confirmed that platelets with suitable concentration might confer resistance to apoptosis by taxane chemotherapy or not. Cancer cells migrate to the vasculature and interact with platelets resulting in tumor cell-induced platelet aggregation during haematogenous metastasis [33]. Co-incubation resulted in platelet aggregation and high concentrations of platelets were more likely to agglutinate. Platelet aggregation around tumor cells provides numerous advantages to the cancer cells. The platelet coating and fibrin (ogen) deposition of tumor cells form a physical barrier to avoid direct contact with NK cells [34]. In addition,platelet aggregation around cancer cells is also essential for the protection of cancer cells against high shear stress in the bloodstream [35].

Here we further demonstrated that platelets of suitable concentration protected against apoptosis in co-culture with SKOV3 cell lines. Tumors induce thrombocytopenia by secreting inflammatory factors, which may also lead to myelosuppression and metabolic disorders in patients [36]. Cancer cells release thromboxane after activating platelets, and high expression of thromboxane receptors and thromboxane synthase have been reported in many cancers. Inhibition of thromboxane synthase activity reduced tumor proliferation saved by the addition of thromboxane A2 and induced apoptosis [37, 38]. Platelets support cancer progression at several levels, particularly at late stages in primary tumors and in metastatic dissemination [39]. However, a few studies demonstrate tumor-suppressive roles at earlier stages, via growth-suppressive effects through downregulation of tumor cell genes and induction of tumor cell apoptosis [40]. The results of this study further complicated the platelet-cancer relationship. Platelets have both positive and negative effects on cancer in different mechanisms and at many stages. Some population and clinical studies have shown that long-term aspirin treatment reduces the mortality of intestinal and non-gastrointestinal cancers and prevents cancer metastasis. It is likely to suggest the role of chronic aspirin in chemoprophylaxis, but forward-looking evidences are still needed. It has been speculated that the binding of platelets to cancer cells protects cancer cells from damage induced by fluid shear stress in blood circulation [35], which can sensitize both colon and prostate cancer cells to tumor necrosis factor-related apoptosis-inducing ligand-induced apoptosis [41]. Inhibition of platelets or related derivatives are expected to increase the sensitivity of cancer cells to apoptosis. Platelet may have a supportive role in docetaxel-induced apoptosis. However, its effect on apoptosis induced by other damage is not clear, and its role in chemoprotection still needs to be further clarified. Aspirin, at a dose of about 100 mg/day (equivalent to a low dose of human), has been given to colorectal cancer mice and the results show that aspirin may induce apoptosis of cancer cells through a mechanism involving down-regulation of IL-6-STAT3 signal pathway [42]. Further, relative thrombocytopenia may be of therapeutic benefit, and within carefully defined safety parameters, the use of anti-platelet reagents may be considered as chemosensitizers.

This retrospective study has various limitations. Firstly, the data were limited by provider variation of plasma D-dimer, fibrinogen, platelet levels and CA125. At the same time, there is no more detailed analysis of different subtypes. Secondly, we did not address a mechanistic relationship between thrombocytosis and tumor apoptosis molecular biology. Lastly, standardization as well as prospective analysis could allow the development of prospective algorithms to test for the predictive value of platelet response as a biomarker for tumor response.

The majority of patients with advanced stage disease EOC will develop recurrence. As we move towards individualized cancer care based on patient specific genome analysis, patient centered outcomes will rise in importance and predictors of recurrence are more important than ever. Platelet level before pretreatment, during the therapy and at the time of recurrence may assist with the ability to predict disease progression and recurrence and may also help identify patients who will benefit from the targeted therapy of cross-talk between platelets and tumor cells. Additionally, the introduction of the convenient prognostic factors such as platelet counts variation tendency can assist clinicians with better individualization of the therapeutic approach of recurrent EOC patient based on the risk stratification.

## Data Availability

The datasets generated during and/or analysed during the current study are available from the corresponding author on reasonable request.

## Abbreviations

EOC: Epithelial ovarian cancer
PFS: Progression-free survival
OS: Overall survival
D-dimer: Dimerized plasmin fragment
PI: Propidium iodide
CA125: Cancer antigen 125
